# An automated platform for *in situ* serial crystallography at room temperature

**DOI:** 10.1107/S2052252520011288

**Published:** 2020-09-19

**Authors:** Zhong Ren, Cong Wang, Heewhan Shin, Sepalika Bandara, Indika Kumarapperuma, Michael Y. Ren, Weijia Kang, Xiaojing Yang

**Affiliations:** a Department of Chemistry, University of Illinois at Chicago, 845 W Taylor St, Chicago, IL 60607, USA; b Renz Research, Inc., Westmont, IL 60559, USA; c A. James Clark School of Engineering, University of Maryland, College Park, MD 20742, USA; d Department of Ophthalmology and Vision Sciences, University of Illinois at Chicago, 845 W Taylor St, Chicago, IL 60607, USA

**Keywords:** *in situ* diffraction, Laue diffraction, monochromatic oscillation, structural dynamics, serial crystallography

## Abstract

Direct observation of functional motions in protein structures is highly desirable for understanding how these nanomachineries of life operate at the molecular level. Because cryogenic temperatures are non-physiological and may prohibit or even alter protein structural dynamics, it is necessary to develop robust X-ray diffraction methods that enable routine data collection at room temperature. We recently reported a crystal-on-crystal device to facilitate *in situ* diffraction of protein crystals at room temperature devoid of any sample manipulation. Here an automated serial crystallography platform based on this crystal-on-crystal technology is presented. A hardware and software prototype has been implemented, and protocols have been established that allow users to image, recognize and rank hundreds to thousands of protein crystals grown on a chip in optical scanning mode prior to serial introduction of these crystals to an X-ray beam in a programmable and high-throughput manner. This platform has been tested extensively using fragile protein crystals. We demonstrate that with affordable sample consumption, this *in situ* serial crystallography technology could give rise to room-temperature protein structures of higher resolution and superior map quality for those protein crystals that encounter difficulties during freezing. This serial data collection platform is compatible with both monochromatic oscillation and Laue methods for X-ray diffraction and presents a widely applicable approach for static and dynamic crystallographic studies at room temperature.

## Abstract 2

An automated platform for diffraction experiments at room temperature for the study of macromolecular structures and dynamics with important biological significance is reported here. This platform preserves the virgin quality of crystal samples and requires low sample consumption.

## 1. Introduction   

Elucidating protein structural dynamics at the molecular level is the key to our fundamental understanding of biochemical reactions and biological processes. As the method of choice, crystallography offers superb spatial and temporal resolution for visualizing protein structures at work. Despite its highly streamlined workflow, protein crystallography has been largely limited to cryogenic conditions that inherently deter large-amplitude protein motions and impede the introduction of structural perturbations for dynamic studies. On the other hand, room-temperature diffraction methods, although highly desirable for dynamic studies, have experienced arrested development over the past decades since cryocrystallography became the gold standard for protein structure determination (Shoemaker & Ando, 2018 ▸). Renewed interests in recent years have been inspired by the great promises of short X-ray pulses from polychromatic synchrotron beamlines and X-ray free-electron lasers (XFELs) in protein dynamics studies (Kupitz *et al.*, 2014 ▸; Nango *et al.*, 2016 ▸; Nogly *et al.*, 2018 ▸; Stellato *et al.*, 2014 ▸; Tenboer *et al.*, 2014 ▸). Surprisingly large conformational changes have been observed at room temperature (Weinert *et al.*, 2019 ▸) as well as elevated cryo temperatures above the glass transition point (Bandara *et al.*, 2017 ▸; Shin *et al.*, 2019 ▸; Zeng *et al.*, 2015 ▸). However, since protein crystals are extremely sensitive to X-ray radiation damage at room temperature, serial protocols must be adopted to collect X-ray diffraction data from a large number of crystals. However, the current serial crystallography platforms face multiple challenges from sample economy to sample integrity, which hinder their wider applicability to many macromolecular systems of great biological and biomedical significance (Fuller *et al.*, 2017 ▸; Wang *et al.*, 2014 ▸; Weierstall, 2014 ▸). We resort to *in situ* diffraction to optimize sample economy and to preserve sample integrity simultaneously. *In situ* diffraction is performed strictly by shooting crystals in their original place where they grew without ever breaking the seal of the crystallization chamber, that is, a procedure devoid of sample mounting, loading, transfer, drying, air exposure or any manipulation.

The conventional crystal mounting techniques such as the loop mounting in cryocrystallography (Teng, 1990 ▸) and the capillary mounting at room temperature (Bernal & Crowfoot, 1934 ▸) require physical manipulation of the specimens, which can be detrimental to protein crystals susceptible to mechanical stress, dehydration, oxidation or unintended activation. The resolution and quality of a diffraction dataset often depend on how well a crystallographer masters the mounting technique. It is difficult, if not impossible, to scale up such a manual operation for serial crystallography applications. Owing to recent developments in injector-based technologies, an astronomical number of nano or microcrystals can be readily introduced into an X-ray beam. However, these delivery systems often suffer from a deficient hit rate and diminishingly minute yield, two metrics defined differently to measure the economy of crystal use (Ren *et al.*, 2018 ▸), which severely limits their applicability to many biological systems. To address this challenge, a variety of fixed-target solutions have been proposed for serial crystallography (Baxter *et al.*, 2016 ▸; Dhouib *et al.*, 2009 ▸; Kisselman *et al.*, 2011 ▸; le Maire *et al.*, 2011 ▸; Perry *et al.*, 2013 ▸; Wierman *et al.*, 2019 ▸) and *in situ* diffraction (Axford *et al.*, 2012 ▸; Bingel-Erlenmeyer *et al.*, 2011 ▸; Sanchez-Weatherby *et al.*, 2019 ▸), among which, we first proposed *in situ* serial Laue diffraction (Perry *et al.*, 2014 ▸). However, implementation of an automated and robust platform for high-throughput serial data acquisition remains a challenging task for large-scale operation. We recently reported a crystal-on-crystal crystallization device to facilitate serial introduction of thousands of the randomly oriented crystals for *in situ* diffraction with little waste of protein crystals (Ren *et al.*, 2018 ▸). Our design features the unconventional use of a monocrystalline substrate such as α-quartz to ensure stable protein crystallization while minimizing background scattering in protein diffraction patterns (Ren, 2017 ▸). Based on this crystal-on-crystal concept, here we report our implementation of an automated platform for *in situ* serial diffraction at room temperature, denoted inSituX. We have conducted high-throughput serial crystallographic experiments using Laue diffraction at a synchrotron. The quality of the datasets and electron-density maps is comparable, or even favorable in some cases, to those obtained from the same crystals with cryocrystallography, which demonstrates the applicability of inSituX platform for both static and dynamic applications at room temperature.

## 2. Results   

**Figure 1:**
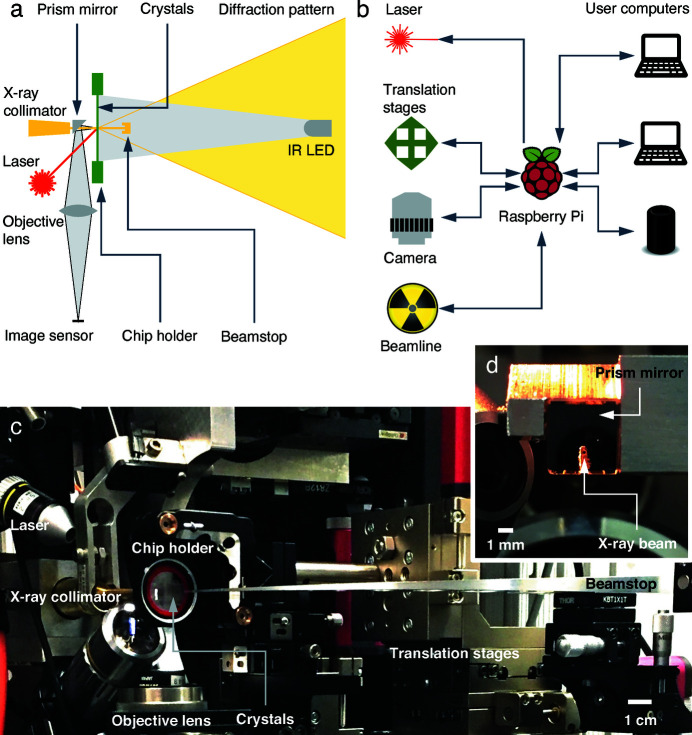
Compact inSituX diffractometer. (*a*) Schematic layout of the diffractometer at the beamline. Each subsystem is colored differently: the crystallization device and motion stages are green, the beamline components for X-ray diffraction are yellow, the optical imaging components are gray and the excitation light is red. (*b*) Schematic flowchart of our control system. At the center of the control system is a Raspberry Pi microcomputer, which coordinates the translation stages that carry samples with the imaging, X-ray and excitation subsystems, and communicates with multiple user computers for data analyses. (*c*) Diffractometer setup installed at the BioCARS 14-ID-B beamline. The diffractometer configured in the data collection mode is shown. (*d*) Micrograph looking into the prism mirror. This view is upstream along the X-ray beam. The final aperture of the X-ray beam is visible through the slot in the mirror. See text for detail.

We have previously shown that a variety of protein samples, including photoreceptors, DNA repair enzymes, oxygen sensors and DNA polymerases, can be crystallized on the crystal-on-crystal chips using the microbatch method (Ren *et al.*, 2018 ▸). This coin-sized device is readily compatible with a standard synchrotron or XFEL beamline with minimal modification. No additional hardware is required if a beamline is equipped with two key capabilities: (1) motorized sample positioning with a travel range of ±12 mm around the X-ray beam in all directions; and (2) an on-axis digital camera for crystal viewing. For those beamlines that do not offer any of these two capabilities, we have developed a compact, portable diffractometer that can be readily accommodated by an existing beamline setup (Fig. 1 ▸).

We have tested a prototype diffractometer on two different beamlines at the Advanced Photon Source (APS): BioCARS 14-ID-B, a polychromatic Laue beamline (Graber *et al.*, 2011 ▸) and LSCAT 21-ID-D, a standard monochromatic beamline equipped with a rapid readout Eiger detector (Dectris Ltd). This diffractometer operates in two modes, optical scanning (Movie S1 of the supporting information) and X-ray diffraction (Movie S2). The main function of the optical scanning mode is to identify and register all qualified protein crystals grown on a quartz device. The X-ray diffraction mode is for serial data collection in which hundreds or thousands of protein crystals are translocated into the X-ray beam one after another at room temperature. Using two rather fragile protein crystals, we demonstrate that this platform enables automated data collection, giving rise to complete crystallographic datasets by combining hundreds or thousands of diffraction images collected from only a few devices. Each image records diffraction from a fresh crystal or a fresh volume of a crystal that has not been previously exposed to X-rays. This *in situ* diffraction platform offers a portable, cost-effective and widely applicable serial crystallography solution for both static and dynamic applications at room temperature.

### 2.1. Diffractometer   

Our prototype diffractometer is built mostly with off-the-shelf components (Thorlabs, Inc.). A three-axis translation stage with a kinematic mount is used to hold and move a quartz device (Fig. 1 ▸). Along two of the three axes perpendicular to the X-ray beam, the stages are motorized to move within a travel range of 25 mm in each direction so that the entire crystallization window of the device is covered. The third axis along the X-ray beam can be adjusted manually. Two manual tilting stages are available to ensure that the quartz chip is perpendicular to the X-ray beam. For monochromatic oscillation, a motorized rotation stage is inserted into the setup with its rotation axis perpendicular to the X-ray beam (see below). For Laue diffraction, no angular motion of the quartz device is required. These motors are controlled by a sequence of instructions encoded in an XML file, a human- and machine-readable markup language. The motor controllers provide electronic signals indicating their motions. These signals are integrated in our control system implemented on a Raspberry Pi microcomputer (Raspberry Pi Foundation) to take advantage of the versatile communication capability of the general-purpose input–output [GPIO; Fig. 1 ▸(*b*)]. The software components of inSituX operate the diffractometer in both optical scanning and X-ray diffraction modes.

In the mode of optical scanning, a 4× microscope objective lens (Nikon) is coupled to an 8 megapixel image sensor of the Pi microcomputer without an infrared (IR) filter. This digital camera captures still images of the on-chip crystals at an adjustable resolution of 1–3 µm pixel^−1^ or feeds a video stream to a monitor or the Internet. A small prism mirror of 5 mm is placed between the quartz chip and the end of the X-ray collimator so that the camera views the crystals on-axis along the incident X-rays [Fig. 1 ▸(*a*)]. A narrow slot of 500 µm cut through one edge of the prism allows the X-ray beam to pass through the prism mirror arriving at the chip for X-ray diffraction [Fig. 1 ▸(*d*)]. At the downstream position of the chip, a light emitting diode (LED) at an IR wavelength of 1085 nm illuminates the chip from a distance of 14 cm. At this wavelength, the IR absorption by water features a local minimum, which alleviates potential heating to protein crystals. Use of IR illumination also prevents unintended photoactivation of photosensitive samples. Since the IR light source and the X-ray beamstop share the same downstream location, they are manually installed one at a time with kinematic mounts depending on the mode of operation. As a result, no live images of the crystals are available during X-ray data collection.

### 2.2. Optical scanning and crystal recognition   

The optical scanning mode is implemented to recognize, locate and rank protein crystals grown on a chip. The crystal positions must be determined with respect to the direct beam position. Therefore, we first install a thin crystal of doped yttrium aluminium garnet (YAG) or an X-ray burn paper at the same position as the crystallization devices and then capture the direct beam fluorescence image or a burn mark on the Pi camera. The accurate position of the direct X-ray beam is obtained by profile fitting. When the same camera settings are used for the optical scanning and direct beam measurement, the crystal positions relative to the X-ray beam can be readily obtained, which will ensure accurate centering of every protein crystal at the X-ray beam for serial diffraction.

A high-resolution image of crystals grown on the device is used for crystal recognition. To obtain this image, a newly installed crystallization device is driven by the translation stages to an array of pre-programmed positions, at which the Pi camera takes IR transmission micrographs of the entire chip (Movie S1). The repetition rate of the translocation is about 1 Hz and the exposure time of each IR micrograph is on the order of milliseconds which does not affect the elapsed time of the optical scan. These IR micrographs are automatically pushed to multiple-user computers for image tiling, crystal recognition and shot planning [Fig. 1 ▸(*b*)].

**Figure 2:**
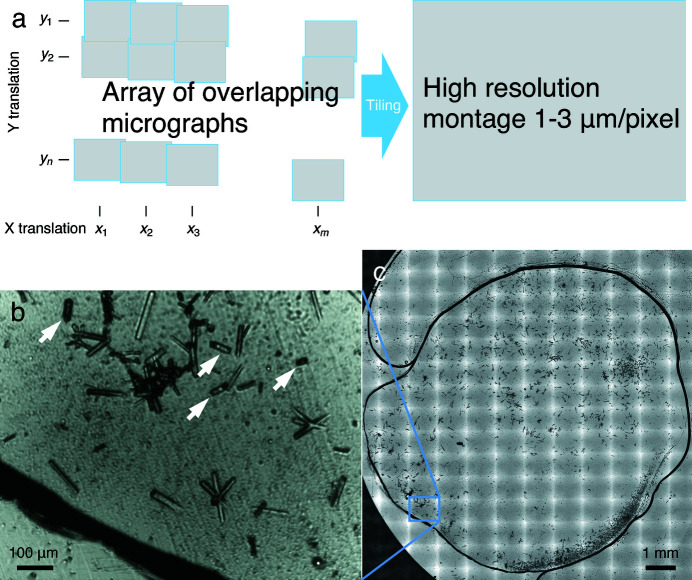
Tiling of sample micrographs. (*a*) Array of overlapping micrographs of the samples is tiled together to produce a high-resolution montage. The translation stages are not necessarily aligned with the edges of the imaging sensor. Possible errors can be corrected. (*b*) Individual transmission micrograph under IR. Several crystals marked by arrows appear shorter from this view. They are actually oriented more perpendicular to the chip compared with the longer ones laying on the chip. These crystals help to fill the entire reciprocal space with observed reflections. (*c*) Montage showing the entire area of the crystallization solution. The micrograph in (*b*) is outlined.

**Figure 3:**
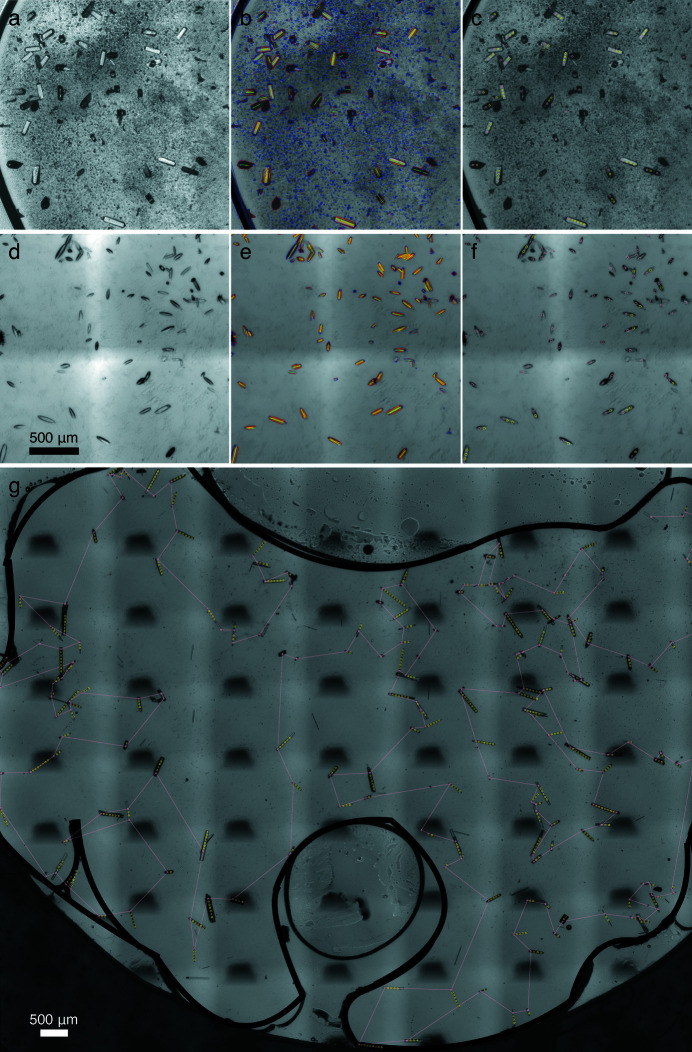
Crystal recognition and shot planning. (*a*) Small portion of a chip with G3 crystals. The darker crystals from this view are more perpendicular to the chip. (*b*) Line segments in various stages of crystal recognition in the same area as (*a*). See text for details. (*c*) Shots automatically planned in the same area as (*a*). The primary shots on each crystal are pink. The subsequent shots are yellow. (*d*)–(*f*) are the same as (*a*)–(*c*) except for Pa497 crystals. (*g*) Traveling salesman solution showing a near-shortest route to visit every crystal once.

Many tens to hundreds of micrographs are taken in a few minutes and are stitched together according to the cross correlation between two adjacent images in the overlapping regions along both the horizontal and vertical directions (Fig. 2 ▸). The resulting montage of hundreds of megapixels at an adjustable resolution of 1–3 µm pixel^−1^ provides a detailed depiction of the crystallization window of 20 × 20 mm on the device [Fig. 2 ▸(*c*)]. If the IR light source is used, images will contain no information of the crystal color. If desired, colored images can be obtained under white light or any light choice of the user. For crystal recognition, the line segment detector of Open Source Computer Vision Library [OpenCV; blue in Figs. 3 ▸(*b*) and 3(*e*)] is used to identify variations in the monochromatic light intensity on the montage photograph. Millions of detected line segments are then filtered by length [red in Figs. 3 ▸(*b*) and 3(*e*)] and paired according to the inter-segment parallelism [yellow in Figs. 3 ▸(*b*) and 3(*e*)]. Since regular-shaped protein crystals are characterized by sharp edges of nearly parallel line segments, they can be readily identified [green in Figs. 3 ▸(*b*) and 3(*e*)]. Crystals with curved edges can be recognized equally well by this algorithm under a user-defined tolerance in parallelism [Figs. 3(*d*), 3(*e*) and 3(*f*)]. For those odd-shaped crystals, deep learning techniques may help to improve the robustness of crystal recognition (Bruno *et al.*, 2018 ▸; Liu *et al.*, 2008 ▸). Once protein crystals are located, X-ray shots are then planned according to the X-ray beam size and the laser beam size [Figs. 3 ▸(*c*) and 3(*f*)]. Overlapping between adjacent X-ray shots is avoided. Collateral laser illumination, including scattering contamination from an adjacent crystal, is controlled by user parameters. Concomitantly, crystal clusters or other irregular objects on the chip arising from precipitation or debris are largely avoided.

The recognized crystals could be ranked by the volume of each crystal or other metrics. We leave a software plugin for a user definition of ranking score. Alternatively, the sequence of targeted shots could follow a traveling salesman solution, a near shortest route to visit each and every targeted crystal once and once only [Fig. 3 ▸(*g*)]. This strategy results in a greatly reduced length of the total motion compared with the sequence that follows the ranking of crystals. We have achieved an analysis of the crystal micrographs within 10–15 minutes, including image tiling, crystal recognition, ranking and target planning, with a single user on a laptop computer. This opportunity for user intervention and decision making improves the overall success of the data collection. We have also achieved an operation frequency of 1 Hz, that is, one X-ray diffraction image per second, which could be improved with more advanced translation stages. Obviously, a near-shortest route and the descending order of the crystal rank are not in agreement. Both options are available to users. Given the pixel coordinates of the direct beam and planned X-ray shots, we can translate each targeted crystal to its corresponding motor positions, which are then encoded in an XML file along with a sequence of instructions to drive the translation stages, and to trigger laser and X-ray shutters for serial diffraction and pump–probe experiments (Fig. 1 ▸).

### 2.3. Monochromatic oscillation   

**Figure 4:**
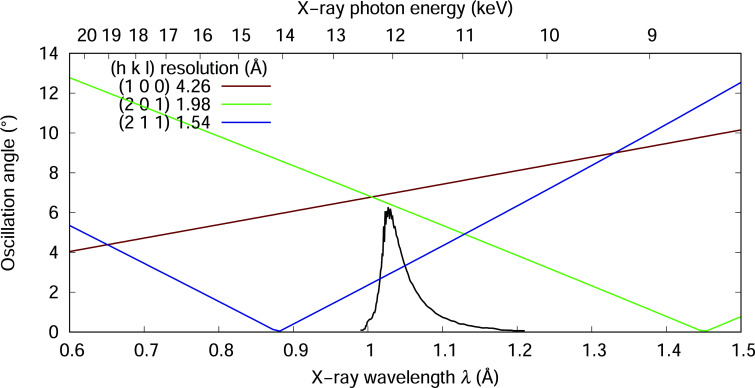
Oscillation limit ω*_hkl_* as function of X-ray wavelength or photon energy. A *Z*-cut α-quartz perpendicular to the X-ray beam can oscillate ±ω*_hkl_* before reflection (*hkl*) occurs. The black curve is a typical wavelength normalization obtained at BioCARS 14-ID-B beamline of APS, which is a good representation of the incident spectrum of the X-ray beam. If a monochromatic wavelength is selected near the peak, the quartz chip can oscillate ±6° without producing a Bragg reflection up to a resolution of 1.54 Å from the quartz crystal.

Although translational motion alone is sufficient for serial Laue data collection, sample oscillation is needed for recording full reflections if a monochromatic X-ray source is used. Given the crystalline nature of the device, it will run into a diffraction condition of the quartz crystal during a rotational motion. However, there are angular gaps where no Bragg diffraction would occur from the quartz crystal because of its small unit cell of ∼5 Å. Therefore, it is important to consider, first, how much a mounted device can oscillate before a Bragg reflection from the single-crystal quartz enters the detector space; and second, which X-ray wavelength (λ) enables the largest possible oscillation without interfering with the protein diffraction. To address these questions, we perform the following calculation. As discussed, three reflections from quartz, (100), (201) and (211), are of the most concern (Ren *et al.*, 2018 ▸). Given a trigonal reciprocal cell of a* = b* = 0.235 Å^−1^ and c* = 0.185 Å^−1^, a *Z*-cut α-quartz wafer may oscillate ±ω*_hkl_* from a starting orientation perpendicular to the X-ray beam before the reflection (*hkl*) occurs. It can be shown that
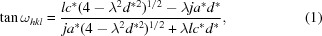
where the reciprocal spacing of the reflection (*hkl*) d* = (*j* ^2^ a*^2^ + 1^2^ c*^2^)^1/2^ . The expression *j* = (*h* ^2^ + *hk* + *k* ^2^)^1/2^ is a discrete function of indices *h* and *k*. Therefore, *j* can be considered as a joint index in planes perpendicular to c*. So we can plot the oscillation limit ω*_hkl_* as function of the wavelength (Fig. 4 ▸). As diffraction from a typical protein crystal rarely exceeds a resolution of 1.5 Å, this plot shows that a ±6° oscillation is allowed at an X-ray wavelength of 1 Å. Such a limit arises from the reflections (100) and (201) of the quartz crystal, as they intercept with the Ewald sphere as the device oscillates in opposite directions. For protein crystals diffracting beyond 1.5 Å, X-rays of 11 keV or 1.13 Å in wavelength would permit an oscillation of ±4°. This analysis shows that the quartz devices allow an oscillation range sufficiently large for monochromatic diffraction without direct interference of any Bragg reflections from the quartz crystal. Due to the limited oscillation, the rotation center of the crystallization device can tolerate a much larger error than that of the normal oscillation method.

To synchronize device oscillation with an X-ray exposure that may last a fraction of second to several seconds, we have incorporated a rotation stage in our diffractometer and developed the required control software. We have also conducted experiments at LS-CAT of APS to test the serial data collection mode using monochromatic oscillation. Compared with Laue diffraction, the achievable time resolution by monochromatic oscillation is at least five orders of magnitude slower at a synchrotron, which may limit its applications for dynamic studies. Alternatively, monochromatic still methods devoid of any angular oscillation, such as those used at XFELs, have to solve the problem of partial integration. Nevertheless, monochromatic methods with or without oscillation may offer an advantage of better spatial resolution in the absence of the polychromatic background scattering.

### 2.4. Serial Laue datasets of bilin-based photoreceptors   

We have developed an automated Laue data collection protocol from the quartz devices with and without light illumination. In this protocol, the translation stages visit every planned target position according to a ranked list or a near-shortest route. Upon completion of every move, the Pi microcomputer sends a trigger signal to fire a succession of laser and X-ray pulses followed by a detector readout. Hundreds and thousands of diffraction patterns can be collected from one quartz device (Movie S2).

We have previously demonstrated the use of the quartz device for *in situ* diffraction (Ren *et al.*, 2018 ▸). Here we report how automation enables large-scale room-temperature serial crystallography studies on two distinct bilin-based photoreceptors. These photosensitive crystals with an ordinary resolution limit do not diffract X-rays very strongly. Their diffraction quality deteriorates quickly upon exposure to room light, presumably because of light-induced conformational changes. They are also extremely sensitive to X-ray radiation. Each X-ray exposure lasts several µs to tens of µs. A longer exposure does not necessarily produce a higher signal-to-noise ratio as the later portion of the exposure contributes only to the background when the crystal is already damaged. Therefore, these crystals represent rather difficult cases compared with robust crystals often chosen as test cases. In this work, we focus on the technical results and will leave the scientific findings for publications elsewhere.

The first system is a novel far-red photoreceptor that confers reversible photoconversion between the far-red-absorbing (Pfr) state and orange-absorbing (Po) state based on a single bilin-binding GAF domain (Bandara *et al.*, 2020 ▸). Since our photoreceptor construct corresponds to the third GAF domain of a multi-domain sensory histidine kinase from *Anabaena cylindrica* PCC 7122, we refer to this crystal as G3 hereafter. Using single-crystal absorption spectroscopy, we are able to confirm the dark-adapted Pfr state in the G3 crystals, which are as photoactive as in solution (Bandara *et al.*, 2020 ▸). Using conventional cryocrystallography, we obtain crystallography datasets of G3 in the space group *P*4_2_22 up to 2.9 Å resolution. Although we are able to determine the crystal structure of G3 in the Pfr state, many important regions are not well resolved in the electron-density map despite our best efforts of trying different ways of freezing using various cryo-protectants. It is likely that the G3 crystals are not amenable to freezing due to the high solvent content of ∼70%.

We therefore resort to the room-temperature method reported here. We are able to crystallize G3 on quartz chips in the dark using the microbatch method. We carry out *in situ* serial diffraction experiments using 22 crystallization devices, from which we obtain a Laue dataset of nearly 50-fold redundancy. Structure refinement against this room-temperature Laue dataset gives rise to a well resolved electron-density map with no gaps in the protein backbone, which enables confident interpretation of a highly unusual and important structure (Table S1 of the supporting information) (Bandara *et al.*, 2020 ▸).

**Figure 5:**
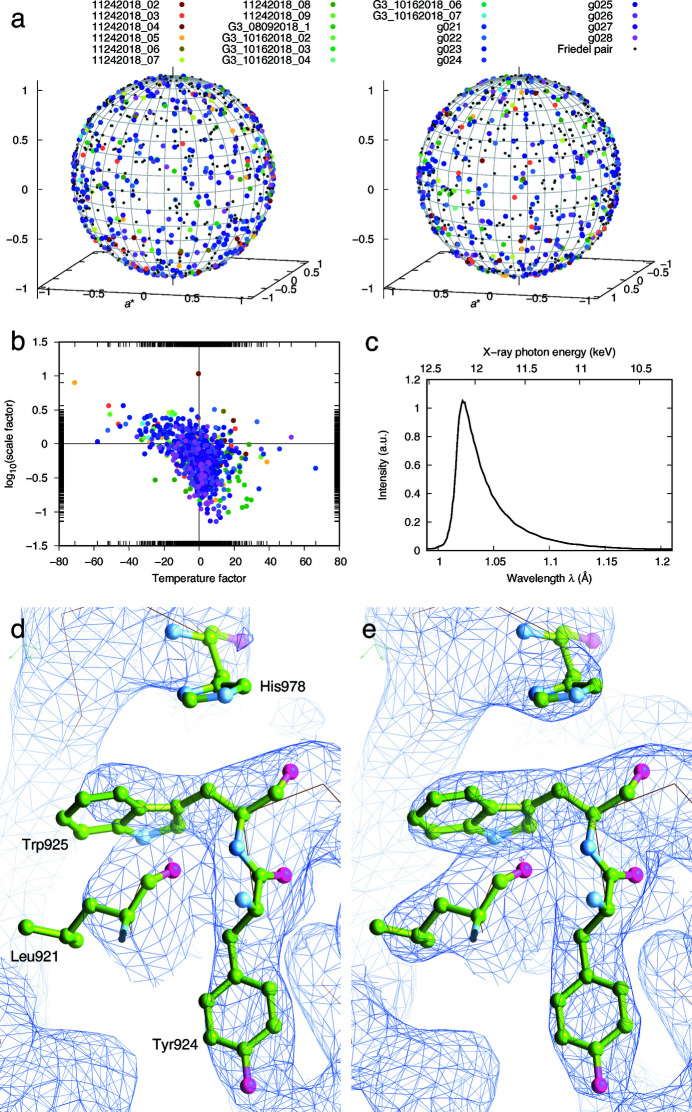
Results from G3 crystals. (*a*) Orientation distribution of 802 G3 crystals. Crystals from different chips are distinguished by color. Smaller black dots indicate the inverted orientations. (*b*) Distribution of frame-to-frame scale factors and temperature factors. (*c*) Wavelength normalization curve extracted from the data. (*d*) and (*e*) Comparison of monochromatic oscillation data at (*d*) 100 K and serial Laue data at (*e*) room temperature. 2*F*_o_ − *F*_c_ electron density maps in the same region are contoured at 1σ. The Laue dataset is an average from many crystals yet defines the conformations of several large side chains better.

In our serial data collection protocol, each crystal or each volume of a larger crystal diffracts the polychromatic X-ray only once [circles in Figs. 3 ▸(*c*) and 3(*f*)]. The exposure time of each frame is 22 µs, which is much shorter than seconds or sub-seconds for monochromatic diffraction. In other words, each diffraction image is the result of the first X-ray exposure of a fresh crystal. More than 4000 crystals are targeted. The final dataset is obtained by merging reflections from more than 800 diffraction images collected at random crystal orientations (Table S1). There appears to be no preferred orientation [Fig. 5 ▸(*a*)]. The diffraction quality seems to vary significantly from crystal to crystal, as evident by the wide distributions in scale factor and temperature factor from one frame to another [Fig. 5 ▸(*b*)]. The wavelength normalization curve derived from the measured spot intensities reveals an X-ray spectrum of a rather narrow bandwidth of 2.7% full width at half-maximum [FWHM; Fig. 5 ▸(*c*)]. It is noteworthy that the total protein consumption is ∼0.8 mg for this dataset derived from 22 devices (see *Methods* [Sec sec4]).

Compared with the best G3 dataset obtained by cryocrystallography using the monochromatic oscillation method, this room-temperature Laue dataset shows superior quality both in electron-density maps (real space) and in refinement statistics (reciprocal space) [Figs. 5 ▸(*d*), 5(*e*) and Table S1]. This can be attributed to four major factors. First, the serial dataset is derived from diffraction patterns captured only at the first moment of X-ray exposure from fresh crystals. Second, with our *in situ* devices, protein crystals are diffracted from the very location where they grew, thereby eliminating any manipulation that may compromise the crystal integrity due to sample transfer, air exposure or mechanical stress, which is in stark contrast to other fixed-target methods that often require exquisite steps of sample manipulation. Third, automation enables a large-scale serial data collection that delivers a high redundancy of ∼50-fold. Lastly, almost all reflections are fully integrated in Laue diffraction given the polychromatic bandwidth. At the same time, ∼800 Laue patterns achieve a redundancy of ∼50 due to the X-ray bandwidth. This last point contrasts with XFEL data which largely depend on redundancy for integration of full reflections.

We also conducted preliminary pump–probe experiments on G3 crystals. We obtained a Laue dataset for the light state of G3 following 250 ms illumination of far-red light at 785 nm. The far-red light emitted from a semiconductor laser diode is focused and trimmed to a focal spot of 500 µm FWHM, a size much bigger than a typical G3 crystal and the X-ray focal spot. To avoid any unintended photoactivation, those crystals too close to higher ranked crystal targets are flagged and ignored during shot planning. Laue diffraction images from 175 illuminated G3 crystals on 10 devices are merged into a final dataset with a redundancy of 7.5. The light induced structural changes are clearly visible in the chromophore region despite the fact that the data quality of this light dataset is not as good as the Pfr dataset (Table S1).

**Figure 6:**
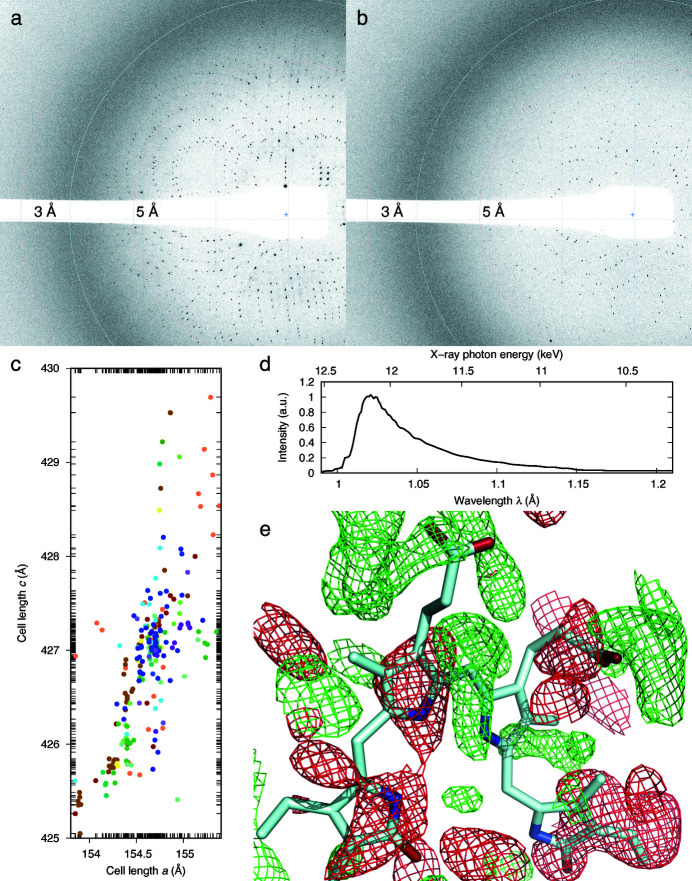
Results from Pa497 crystals. (*a*) One of the best and (*b*) one of the worst diffraction images included in the final dataset. The circular bands mark the wavelength-dependent resolutions at 3 and 5 Å due to polychromatic diffraction. (*c*) Distribution of the refined unit-cell lengths *a* versus *c*. Crystals from different chips are distinguished by color. (*d*) Wavelength normalization curve. (*e*) Difference Fourier map around the bilin chromophore. Green and red meshes are contoured at ±2σ levels.

In the second test case, we study the photosensory core module (PCM), denoted Pa497, of a bacteriophytochrome from *Pseudomonas aeruginosa* (Yang *et al.*, 2008 ▸, 2011 ▸). This highly photosensitive crystal form contains four photoactive dimeric Pa497 molecules in the asymmetric unit (ASU), which renders a much larger molecular mass (460 kDa per ASU) compared with that of the G3 crystal (21 kDa per ASU; Table S1). We crystallized Pa497 on quartz chips using a modified protocol based on the published conditions (see *Methods* [Sec sec4]). By merging 261 diffraction patterns collected from 15 crystallization devices (Movie S3), we obtain a near-complete room-temperature dataset of Pa497 at 3.0 Å resolution with a redundancy of 7.3. Like G3, the Pa497 crystals vary significantly in diffraction quality primarily due to variations in crystal size [Figs. 6 ▸(*a*) and 6(*b*)]. This crystal form also features a cell length along the *c* axis as large as 427.2 Å, which poses certain difficulties to data reduction due to spatial overlaps among crowded diffraction spots. The refined cell parameters show a 1.2% variation (∼5 Å) in *c*, which potentially contributes to the non-isomorphism among crystals (Ren *et al.*, 2018 ▸). Such variation seems independent of the crystallization device [Fig. 6 ▸(*c*)].

We also collected a preliminary light dataset of Pa497 after 500 ms illumination of far-red light at 785 nm emitted from the same laser diode used for the G3 experiments. Laue diffraction images from 151 Pa497 crystals on 12 devices are merged into a dataset with a redundancy of 4.4 (Table S1). Despite the inferior data quality, the difference Fourier map shows significant light-induced structural changes on the bilin chromophore [Fig. 6 ▸(*e*)] which developed far beyond those previously seen at cryo temperatures (Yang *et al.*, 2011 ▸). The total protein consumption for Pa497 dark and light datasets is ∼2 mg (see *Methods* [Sec sec4]). The scientific findings on the sensory histidine kinase and the bacteriophytochrome will be presented elsewhere.

## 3. Discussion   

How to effectively deliver a large number of crystal samples to the X-ray beam is at the center of technological development for serial crystallography at both synchrotrons and XFELs (Grünbein & Kovacs, 2019 ▸; Martiel *et al.*, 2019 ▸). The sample delivery methods largely fall into two categories, injector and fixed target. A commonly used metric to evaluate the efficiency of a sample delivery method is the hit rate, which is defined as the percentage of the hit images, in which diffraction signals are observed, out of the total number of images taken. Given that protein crystals are much more precious than X-ray pulses, we proposed a different metric, called ‘diffraction yield’, to evaluate sample delivery in serial crystallography. The diffraction yield differs from the hit rate in the denominator, in which the total number of prepared crystals replaces the total number of images taken (Ren *et al.*, 2018 ▸). Both the hit rate and the diffraction yield may have their effective forms that measure the fractions of useful images successfully indexed and merged into a final dataset.

Compared with injector-based methods, the fixed-target methods by design have higher hit rates, thus better sample economy: a critical factor in serial crystallography. However, many fixed-target methods reported in the literature entail exquisite crystal manipulation in one form or another as samples are transferred from where they grew onto a carrier for diffraction. While these sample-loading procedures may seem harmless to robust crystals, they inevitably subject fragile protein crystals to potential mechanical stress, dehydration, oxidation or light exposure, which compromise the diffraction quality and limit the applicability of these methods. In contrast, our *in situ* diffraction platform requires no crystal manipulation of any kind. In other words, all protein crystals diffract X-rays at their virgin state. Thus, the probability of obtaining useful diffraction images is significantly improved. *In situ* Laue patterns show little streakiness in the diffraction spots [Figs. 6 ▸(*a*) and 6(*b*); Movie S1] unlike the old-fashioned capillary mounting of protein crystals that often led to radial streaks in Laue images due to the extreme sensitivity of Laue geometry to crystal mosaicity (Ren *et al.*, 1999 ▸). Therefore, this *in situ* platform is a significant improvement to Laue diffraction.

Since hundreds and thousands of protein crystals grown on a chip can appear at any location within the crystallization window, this automated *in situ* diffraction platform reported here is necessary for a large-scale operation. Our implementations of optical scanning, crystal recognition, target planning and high-throughput serial data collection are integrated in the hardware and software components of the inSituX platform. With this automated platform, it is readily possible to conduct an overkill so that no crystal is left unexposed on a chip regardless of how small or how clustered some of the crystals are, that is, 100% yield. Such a brute force strategy may not be the most effective or desirable, since not all crystals will produce useful images. Given that protein crystals are ranked according to their size, shape and clustering status, users may apply a cutoff in such a ranked list so that only those top-ranked crystals are targeted for diffraction. Often, a lower diffraction yield would greatly increase the effective hit rate. We have implemented specific features in the target-planning software of inSituX to allow users to selectively target the desired crystals and exclude the problematic crystals by automated procedure and visual inspection so that an optimal balance can be achieved between the effective hit rate and the diffraction yield. The reported datasets in this work reach effective hit rates ranging from 5 to 20% (Table S1), which could be improved if a lower diffraction yield is sought, that is, higher selectivity during target planning.

This *in situ* fixed-target technology with automation opens up a number of new opportunities for protein crystallography and structural biology. First, this *in situ* diffraction platform is advantageous for fragile crystals that are susceptible to freezing, touching, dehydration or air exposure. Devoid of any crystal manipulation, this technology also ensures the crystal lattice integrity, thereby the best possible diffraction resolution. *In situ* diffraction at room temperature could be an attractive alternative for static structure determination from sensitive crystals in the future. Second, time-resolved studies at synchrotrons have been restricted to extremely robust crystals like myoglobin (Schotte *et al.*, 2003 ▸; Šrajer *et al.*, 2001 ▸) and photoactive yellow protein (Jung *et al.*, 2013 ▸; Schotte *et al.*, 2012 ▸) which can sustain many X-ray exposures to complete a dataset. Dynamic crystallography experiments at XFELs require an astronomical number of microcrystals injected into the X-ray beam for recording of full reflections. Such high demands on sample quality and quantity have hindered wide applications of dynamic crystallography for many important biological systems. Our *in situ* diffraction platform has begun to address these challenges. A robust technology at room temperature, widely applicable to systems of ordinary crystal quality and affordable protein consumption, will lead protein crystallography towards dynamic studies at atomic resolution. This direction of protein crystallography could prove important in light of the recent success of cryoelectron microscopy. Third, given the light and X-ray transparency of the quartz device, this technology is well suited for capturing light-induced structural changes in photoactive proteins. However, to capture ligand-induced structural changes, future developments are required to convert the crystal-on-crystal chip to a microfluidic device by chemical etching (Ren *et al.*, 2018 ▸), which will enable a controlled introduction of a chemical trigger to protein crystals grown on a chip. The results presented in this work demonstrate the feasibility, affordability and thus the wide applicability of a high-throughput *in situ* diffraction platform, which will transform how static and dynamic crystallography are conducted at room temperature.

## 4. Methods   

### 4.1. Crystallization   

The construct of the third GAF domain of a multi-domain sensory histidine kinase from *Anabaena cylindrica* PCC 7122 is co-expressed in the *E. coli* BL21(pLys) strain with a plasmid that carries the heme oxygenase (HO1) and biliverdin reductase genes to produce phyco­cyano­bilin (PCB). After cell lysis, the supernatant is clarified by Talon Co^2+^ affinity chromatography followed by anion-exchange chromatography (5 ml HiTrap HP Q column, GE Healthcare). The fractions with good chromophore incorporation are further purified using size-exclusion chromatography (10/300GL Superdex 200 Increase, GE Healthcare). A microbatch method is used to set up the on-chip crystallization. Specifically, the crystallization solution contains 2 mg ml^−1^ protein, 8.5–10.5% polyethyl­ene glycol 10 000 (PEG), 50 m*M* ammonium acetate and 50 m*M* bis-Tris buffer at pH 5.5. A total volume of 18 µl crystallization solution is loaded into the sealed chamber of a chip assembled with a shim of 125 µm thickness. All devices are incubated and stored in the dark at room temperature. Crystal growth is monitored under a microscope with a filtered safety light at 500 ± 30 nm. Crystals reach a full size of >100 µm within a few days. The total protein consumption for 22 devices is about 0.8 mg.

Pa497 protein is purified as previously described (Yang *et al.*, 2008 ▸). In a microbatch setup, 10 µl crystallization solution is loaded into a crystallization chamber with a thickness of 120 µm. Each microbatch contains 5.0–7.5 mg ml^−1^ of protein, 0.2–0.3 *M* of (NH_4_)_3_PO_4_ and 50 m*M* of Tris buffer at pH 7.7. With lower protein concentration, fewer and larger crystals can be obtained. The crystals can grow to their full size of >100 µm in two weeks. The total consumption of protein for 27 devices is <2 mg.

### 4.2. Monochromatic oscillation data collection and structure determination   

G3 in the Pfr state is crystallized following pre-illumination with filtered green light at wavelengths of 550 ± 20 nm (Newport) for 15 min before setting up the crystallization (Bandara *et al.*, 2020 ▸). The Pfr crystals grow in the dark at room temperature using the hanging-drop vapor-diffusion method by mixing the protein solution (3 mg ml^−1^) and crystallization solution (17% PEG 10 000) and 0.1 *M* ammonium acetate in the buffer of 0.1 *M* bis-Tris at pH 5.5 with yttrium (III) chloride as an additive. Green Pfr crystals of a typical size of 50 × 50 × 100 µm appear within 2–3 days and are harvested with cryo-protectant under a green safety light at wavelengths of 500 ± 20 nm. The monochromatic X-ray diffraction datasets at 100 K are collected at the LS-CAT beamlines of the Advanced Photon Source (APS), Argonne National Laboratory. All diffraction images are processed using *HKL*2000. Initial phases are obtained for the Pfr structure in the space group *P*4_2_22 by molecular replacement (*Phaser*; McCoy *et al*., 2007 ▸) using a homologous structure as a search model (PDB entry 4glq; Burgie *et al.*, 2013 ▸).

### 4.3. Laue data collection and processing   

Laue diffraction experiments are conducted at the BioCARS beamline 14-ID-B of APS; see the methods in the work by Ren *et al.* (2018 ▸) for details. Diffraction images are recorded by a Rayonix MX 340 X-ray detector (Rayonix, LLC). The dark datasets of G3 and Pa497 are collected at partially closed white beam slits to reduce the X-ray power due to a fast chopper out of service. As a compensation, the exposure time of each frame is increased to 22 µs FWHM, that is, 37 µs end-to-end. Accepting only the central cone of the white beam is likely to reduce the bandwidth to ∼3% compared with the usual bandwidth of ∼6% (Ren *et al.*, 2018 ▸). Laue diffraction images are processed with the software package *Precognition/Epinorm* (Renz Research Inc.).

### 4.4. Structural refinement   

The G3 structures at the Pfr state are refined against the Laue and cryo monochromatic datasets by *phenix.refine* (Adams *et al.*, 2010 ▸; https://www.phenix-online.org) (Table S1). The refined structures are deposited in the Protein Data Bank under the accession codes: 6uv8 (Laue at room temperature) and 6uvb (monochromatic oscillation at 100 K).

### 4.5. Software packages and databases   

The following software packages and databases are used in this work: *Coot* (Emsley & Cowtan, 2004 ▸; https://www2.mrc-lmb.cam.ac.uk/personal/pemsley/coot); *HKL*2000 (Otwinowski & Minor, 1997 ▸; https://hkl-xray.com); OpenCV (https://opencv.org); *PHENIX* (Adams *et al.*, 2010 ▸; https://www.phenix-online.org); *Precognition*/*Epinorm* (Renz Research Inc.); Protein Data Bank (https://www.rcsb.org); *PyMOL* (https://pymol.org); Python (https://www.python.org), and SciPy (https://www.scipy.org).

## Supplementary Material

Supporting information file. DOI: 10.1107/S2052252520011288/ti5017sup1.mp4

Supporting information file. DOI: 10.1107/S2052252520011288/ti5017sup2.mp4

Supporting information file. DOI: 10.1107/S2052252520011288/ti5017sup3.mp4

Supporting information file. DOI: 10.1107/S2052252520011288/ti5017sup4.pdf

PDB reference: Laue structure of G3 at room temperature, 6uv8

PDB reference: monochromatic oscillation structure of G3 at 100 K, 6uvb
