# Supplementary material for: An automated platform for in situ serial crystallography at room temperature
Source: IUCrJ. 2020 Sep 19;7(Pt 6):1009–18. doi: 10.1107/S2052252520011288 (PMC7642789; doi:10.1107/S2052252520011288)
Supplement: Supplementary file 4 [file m-07-01009-sup4.pdf]

IUCrJ

Volume 7 (2020)

Supporting information for article:

An Automated Platform for *in Situ* Serial Crystallography at Room Temperature An Automated Platform for *in Situ* Serial Crystallography at Room Temperature

Zhong Ren, Cong Wang, Heewhan Shin, Sepalika Bandara, Indika Kumarapperuma, Michael Y. Ren, Weijia Kang and Xiaojing Yang

Table S1 Data processing and structural refinement

Dataset/PDB ID	G3 Pfr 100K/6uvb	G3 Pfr/6uv8	G3 @ 785 nm	Pa497 dark	Pa497 @ 785 nm
Space group	$P4_222$	$P4_222$	$P4_222$	$C222_1$	$C222_1$
Unit cell (Å)	108.8, 108.8, 68.7	108.8, 108.8, 67.9		154.7, 163, 427.2	
Molecular mass/asymmetric unit (kDa)	21	21	21	460	460
Crystallization devices	-	22	10	15	12
Exposure time per frame (μs)	5 s/1° frame	22	8	22	8
Frames used/collected = index rate (%)	100/100 = 100	802/4,055 = 19.8	175/1,120 = 15.6	261/2,627 = 9.9	151/2,762 = 5.5
Spots measured	-	586,816	85,518	730,280	420,917
Spots merged/spots per frame	62,940/629	577,282/720	84,140/481	716,508/2,745	398,212/2,637
Unique reflections/redundancy	9,394/6.7	11,702/49.3	11,160/7.5	98,518/7.3	90,979/4.4
Harmonic/spatial overlap deconvolutions	-	2,794/165	2,228/40	19,584/1,069	23,383/733
Resolution/high resolution bin (Å)	2.95/3-2.95	2.7/2.8-2.7	2.8/3.0-2.9	3.0/3.1-3.0	3.0/3.1-3.0
Completeness/high resolution bin (%)	98.9/100	99.1/94.2	89.4/45.4	95.1/80.3	85.6/79.7
X-ray bandwidth in FWHM[1] (%)	Single wavelength	2.7	5.6	3.4	4.7
$\langle F \rangle / \sigma(F)$/high resolution bin	30.2/1.0	24.3/11.3	9.7/8.2	10.5/11.0	21.7/23.6
R_{merge} (%)	3.3	22.2	22.7	21.9	9.9
$R_{1/2}$ (%)/$CC_{1/2}$	-	8.7/0.9868	24.4/0.7918	22.0/0.6641	18.2/0.5059
R_{work}/R_{free} (%)	25.8/30.3	21.1/26.1	-	-	-
Bond length/angle rmsd (Å/°)	0.008/1.248	0.015/1.606	-	-	-
Favored/allowed/disallowed Ramachandran (%)	96.1/3.3/0.55	91.8/8.2/0.0	-	-	-

[1]Full width at half maximum

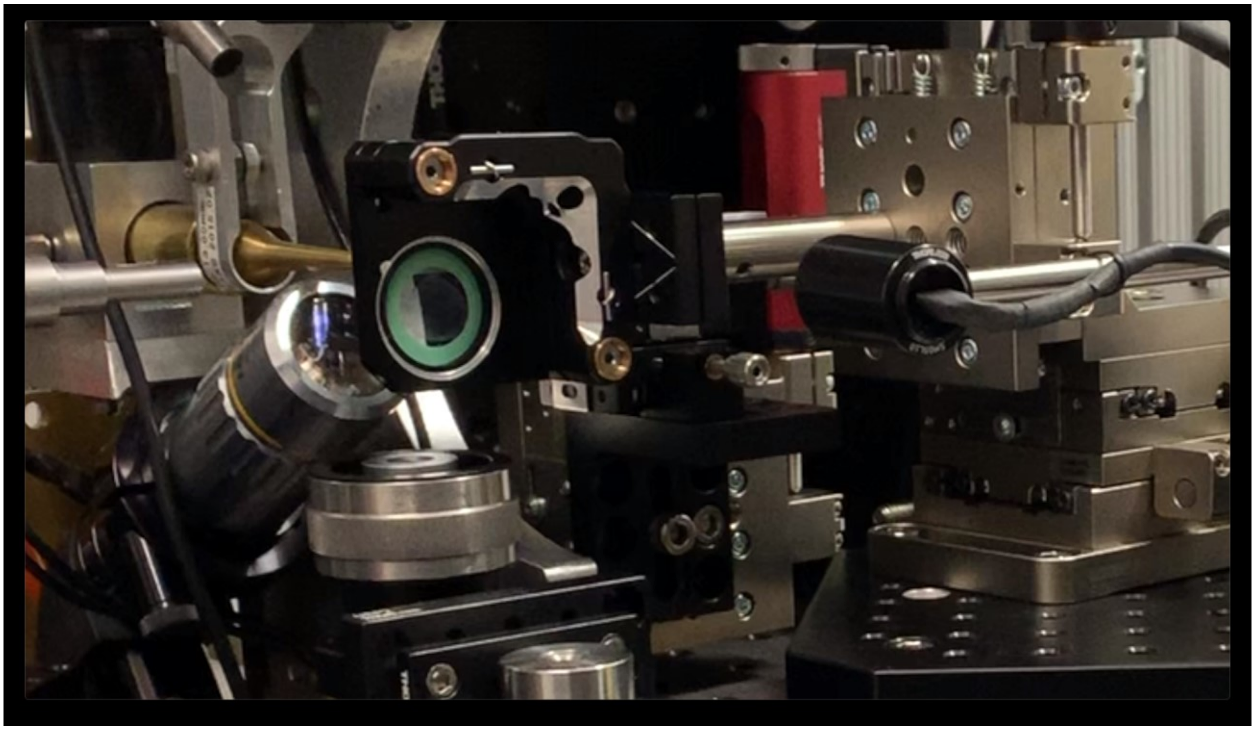

Movie S1. Optical scanning. Crystals grown on a quartz chip (green circle) are being scanned under infrared light. The room light should be off if the crystals are photosensitive during a normal operation. See Fig. 1 legend for detail.

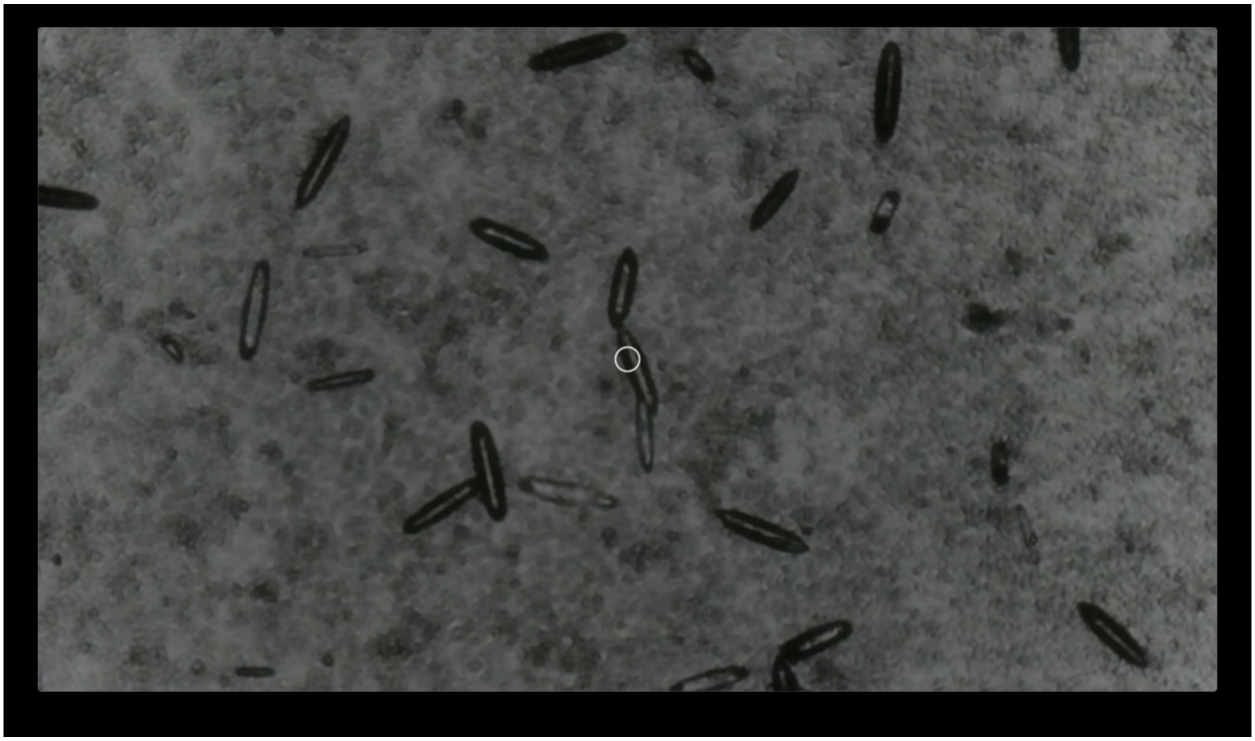

Movie S2. Simulated data collection for X-ray diffraction. The targeted Pa497 crystals are translocated into the X-ray beam (white circle of 30 μm in diameter) in a pre-programed sequence according to a crystal ranking. An X-ray pulse is fired at each stop.

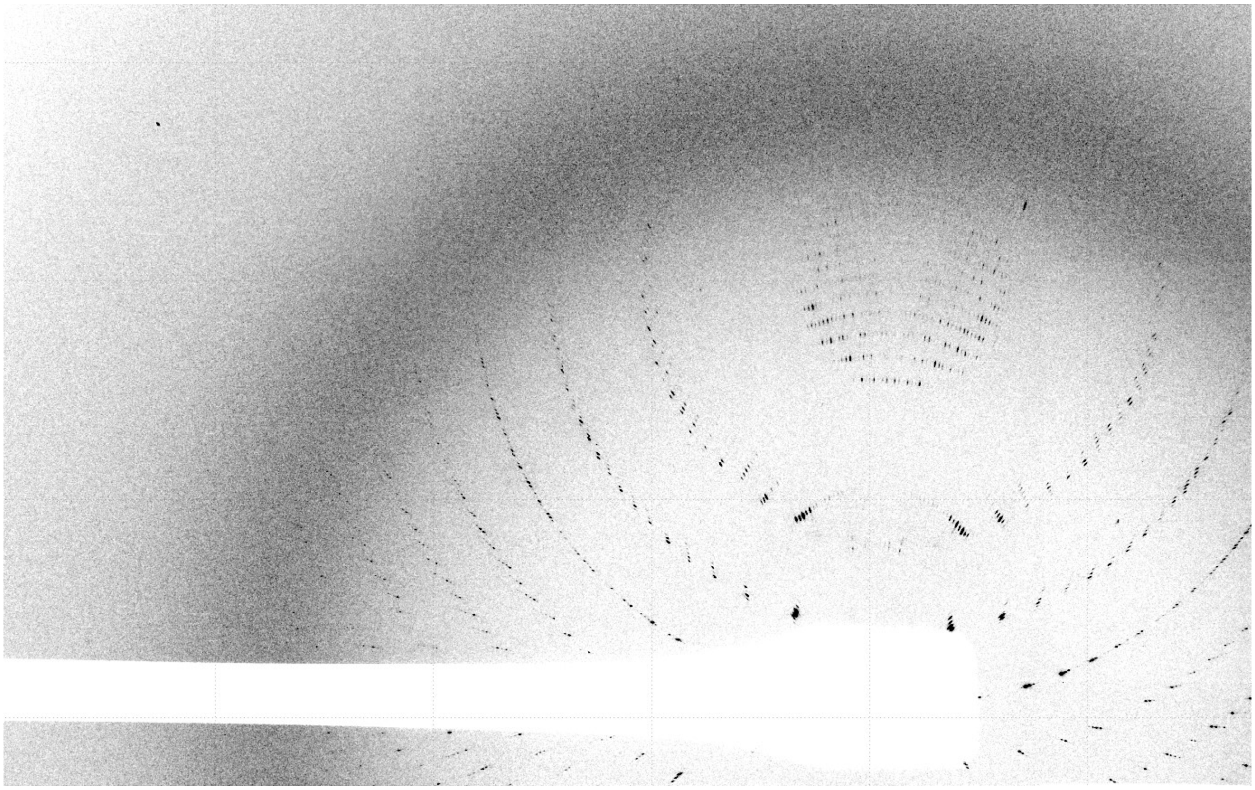

Movie S3. Laue diffraction patterns from Pa497 crystals. A portion of the Laue images at random orientations are shown. Each diffraction image is obtained from a fresh volume of a crystal.
